# The Efficacy of Acupuncture for the Treatment of Sciatica: A Systematic Review and Meta-Analysis

**DOI:** 10.1155/2015/192808

**Published:** 2015-09-06

**Authors:** Mei Ji, Xiaoxia Wang, Meijuan Chen, Yan Shen, Xu Zhang, Jin Yang

**Affiliations:** ^1^College of Basic Medicine, Nanjing University of Chinese Medicine, Nanjing 210023, China; ^2^Jiangsu Collaborative Innovation Center of Traditional Chinese Medicine (TCM) Prevention and Treatment of Tumor, Nanjing University of Chinese Medicine, Nanjing 210023, China

## Abstract

*Background*. Sciatica is one of the most frequently reported complaints; it affects quality of life and reduces social and economic efficacy. Clinical studies on the efficacy of acupuncture therapy in sciatica are increasing, while systematic reviews assessing the efficacy of acupuncture therapy are still lacking. *Objective*. This study aims to assess the effectiveness of acupuncture therapy for sciatica. *Methods*. Comprehensive searches of 8 databases were conducted up until April 2015. Outcomes included effectiveness (proportion of patients who improved totally or partly in clinical symptoms), pain intensity, and pain threshold. Effect sizes were presented as risk ratio (RR) and mean difference (MD). Pooled effect sizes were calculated by fixed effects or random effects model. *Results*. A total of 12 studies (involving 1842 participants) were included. Results showed that acupuncture was more effective than conventional Western medicine (CWM) in outcomes effectiveness (RR 1.21, 95% CI: 1.16–1.25), pain intensity (MD −1.25, 95% CI: −1.63 to −0.86), and pain threshold (MD: 1.08, 95% CI: 0.98–1.17). Subgroup and sensitivity analysis found that the results did not change in different treatment method and drug categories substantially. The reported adverse effects were acceptable. *Conclusions*. Acupuncture may be effective in treating the pain associated with sciatica.

## 1. Introduction

Sciatica is a syndrome rather than a specific diagnosis [1]. In 90% of cases, sciatica is caused by a herniated disc with nerve-root compression [1]. The prevalence ranges from 1.2% to 43% [2]. A number of risk factors are thought to be associated with first-time incidence of sciatica and influence the development of sciatica; these include smoking, obesity, occupational factors, health status, age, gender, and social class [3, 4]. According to traditional Chinese medicine (TCM), sciatica belongs to the gallbladder meridian of the foot-Shaoyang (GB) and the bladder meridian of the foot-Taiyang (BL), and the Yanglingquan (GB 34) and Huantiao (GB 30) are two key “acupuncture points” (acupoints) for treating sciatica [5].

Acupuncture is widely used in clinical practice in China and in many Western countries [6]; in China, it can be traced back at least 3000 years as part of the healing system based on the principles of TCM. Traditional acupuncturists understand health in terms of a vital force or energy called “qi” which circulates between the organs along channels called meridians [7]. Since sciatica is a channel disorder, acupuncture of points removing channel obstruction and promoting qi and blood circulation is indicated in its treatment [8]. Acupuncture points can be stimulated for about 30 minutes by surface pressure, insertion of a needle with or without manipulation, heating of acupuncture needles through radiant heat or moxibustion, and electrical or laser stimulation [9].

Since the early 1990s, original studies [10–12] have reported the efficacy of acupuncture for the treatment of sciatica. However, the results had been controversial. It is relevant to query whether the effectiveness of acupuncture is evident and knowledge of effective interventions is critical in order to reduce the health and safety risks [13]. Up to now, there is only a systematic review protocol [14] about acupuncture for treating sciatica and no published meta-analysis of the effectiveness of acupuncture compared with medication for sciatica; we carried out a comprehensive and quantitative evaluation analysis to assess its efficacy and safety in the clinical treatment of this condition.

## 2. Materials and Methods

### 2.1. Database and Search Strategy

Three Chinese language databases (China National Knowledge Infrastructure (CNKI), Chinese Biomedical Literature Database (CBM), and Wanfang Data) and five English language databases (Cochrane Library, PubMed, Web of Science, Science Direct, and FMRS (Foreign Medical Literature Retrial Service)) were extensively searched until April 30, 2015. Neither the publication status of the search trials nor the basic standard of selecting points for needle insertion was restricted. The search strategy includes the following group terms: English (“acupuncture” OR “electroacupuncture” OR “needle warming therapy” OR “needling methods” OR “fire-needle therapy” OR “acupuncture therapy” OR “acupuncture points” OR “acupuncture analgesia”) AND (“sciatica” OR “sciatic pain” OR “neuralgias, sciatic” OR “sciatic neuralgias” OR “sciatica, bilateral” OR “bilateral sciatica” OR “bilateral sciaticas” OR “neuralgia, sciatic” OR “sciatic neuropathy” OR “sciatic nerve diseases”); Chinese (“zhen jiu” OR “wen zhen” OR “huo zhen liao fa” OR “zhen ci liao fa” OR “zhen jiu zhi liao” OR “zhen jiu xue wei” OR “zhen ci zhen tong” OR “dian zhen”) AND (“zuo gu shen jing tong” OR “zuo gu shen jing bing” OR “zuo gu shen jing yan” OR “zuo gu shen jing ji bing”).

### 2.2. Inclusion and Exclusion Criteria

Studies that met the following criteria were included in the review: (1) studies published in English or Chinese language; (2) randomized or quasi-randomized clinical trials; (3) participating patients that must have been diagnosed with sciatica or presented with any or all of the following symptoms: radiating pain in the sciatic nerve distribution area, tenderness at the nerve stem, positive Lasegue's sign, Kernig's sign, and Bonnet's sign; (4) any of the manual, warm, electric, or laser types of acupuncture that were used; (5) considering the following comparisons: acupuncture versus conventional Western medicine (CWM); CWM are conservative treatments of Western medicine, including oral drugs (e.g., Prednisone, Ibuprofen, and Nimesulide), external drugs (e.g., Diclofenac Diethylamine gel), and injection (e.g., anisodamine); (6) any of the following outcome measures that were eligible: effectiveness, pain intensity, and pain threshold.

Studies that met the following criteria were excluded: (1) randomized crossover trials, case reports, case series, reviews, qualitative studies, or animal experiments; (2) participants with back pain or low back pain but no symptoms of sciatica; (3) interventions that included a combination of more than one treatment strategy (or mixed treatments); (4) studies comparing interventions grouped under the same treatment strategy (e.g., a comparison between different forms or different acupoints of acupuncture).

### 2.3. Definitions

The primary outcome analysis for this meta-analysis was effectiveness (the proportion of patients who improved totally or partly in clinical symptoms). The effectiveness was presented by using the following formula: rate (effectiveness) = (*N*1 + *N*2 + *N*3)/*N*, where *N*1, *N*2, and *N*3 are the number of patients cured, markedly improved, and improved and *N* is the sample size. Criteria for improvement after treatment are the following: cured: all the symptoms and physical signs referred to above disappeared after the treatment with no relapse found in half a year and the patients could resume work; markedly improved: all the symptoms and physical signs basically disappeared but sometimes might relapse or even be more serious and the patients were able to do light work; improved: the symptoms were relieved with improved limb functions, but the pain always recurred.

Pain intensity and pain threshold were considered as the secondary outcomes. Pain intensity, is got by using a visual analogue scale (VAS) to measure pain on a continuous scale with data converted to a scale of 0–100 mm (0 means no pain, 100 means severe pain, and middle section shows different levels of pain). And the VAS is a common means of measuring individuals' rating of their own health [23]. Pain threshold, a threshold value, is got by using a pain measurement instrument to measure potassium ion. The big difference between the two outcomes is that values of the former are subjective (self-assessment by participants), while the latter ones are objective (tested by detector).

### 2.4. Data Extraction

Two reviewers (MJ and YS) independently screened the title and abstract of each searched article for eligibility and relevance. Potentially relevant papers were retrieved for further assessment according to the inclusion and exclusion criteria. One reviewer (MJ) extracted the data and another (YS) checked it, and any discrepancy was resolved by discussion. A standardized form was used for data input, consisting of contents such as general information (first author, publication year), patient characteristics, diagnostic criteria, study design, treatment protocol, outcome measurements (effectiveness, pain intensity, and pain threshold), withdrawal, and adverse events. The aforementioned data of *N*1, *N*2, *N*3, and *N* of effectiveness were also extracted.

The extraction of details of acupuncture treatment and control interventions of studies was on the basis of STRICTA (Standards for Reporting Interventions in Clinical Trials of Acupuncture) reporting guidelines [24] which could improve the completeness and transparency of reporting of interventions in controlled trials of acupuncture. A checklist included acupuncture rationale, details of needling (points used, depth of insertion, response sought, needle stimulation, needle type, and retention time), treatment regimen (number of treatment sessions, frequency), and control interventions.

### 2.5. Quality Assessment

We analyzed the studies using the Cochrane Handbook, Version 5.1.0. [25]. Quality of the included trials was assessed according to seven domains: random sequence generation (selection bias), allocation concealment (selection bias), binding of participants and personnel (performance bias), binding of outcome assessment (detection bias), incomplete outcome data (attrition bias), selective reporting (reporting bias), and other potential sources of bias. Each domain was classified as “yes” (low risk of bias), “no” (high risk of bias), or “unclear” (uncertain risks). This was independently evaluated by two reviewers (MJ and YS). Disagreements were resolved by a third reviewer (XW).

### 2.6. Data Synthesis and Analysis

We used the Cochrane Collaboration Review Manager software (RevMan 5.3) for statistical analysis. The extracted data were classified into dichotomous and continuous variables. Data were summarized using risk ratio (RR) with 95% confidence intervals (CI) for dichotomous outcome; mean difference (MD) with 95% CI was presented for continuous outcome. Heterogeneity across studies was informally assessed by visually inspecting forest plots and formally estimated by Cochran's *Q* test in which chi-square distribution is used to make inferences regarding the null hypothesis of homogeneity (*P* < 0.10 was considered to be representative of statistically significant heterogeneity). We also quantified the effect of heterogeneity using the *I* ^2^ statistic, which measures the degree of inconsistency in the studies by calculating what percentage of the total variation across studies is due to heterogeneity rather than by chance. *I* ^2^ values of 25, 50, and 75% were nominally assigned as low, moderate, and high estimates, respectively [26]. A fixed effects model was used when there was no significant heterogeneity (*I* ^2^ < 50%) of the results of the studies. Otherwise, the random effects model was used (*I* ^2^ ≥ 50%). Based on different outcome measures, we would investigate possible causes from clinical perspectives by conducting subgroup and sensitivity analysis. Various subgroup analyses were performed based on types of route of medication (e.g., oral drugs, external drugs, and injection) and drug categories (e.g., Nimesulide, Indomethacin, and Ibuprofen + Prednisone). Sensitivity analysis was performed by removing each study in sequence and recalculating the results, aiming to assess whether one or more studies influenced the overall results. We used funnel plots to examine asymmetry for publication bias, revealing an asymmetrical distribution of studies around the line of identity, indicating the possibility of a small indistinct study bias [27].

## 3. Results

### 3.1. Study Selection

**Figure 1:**
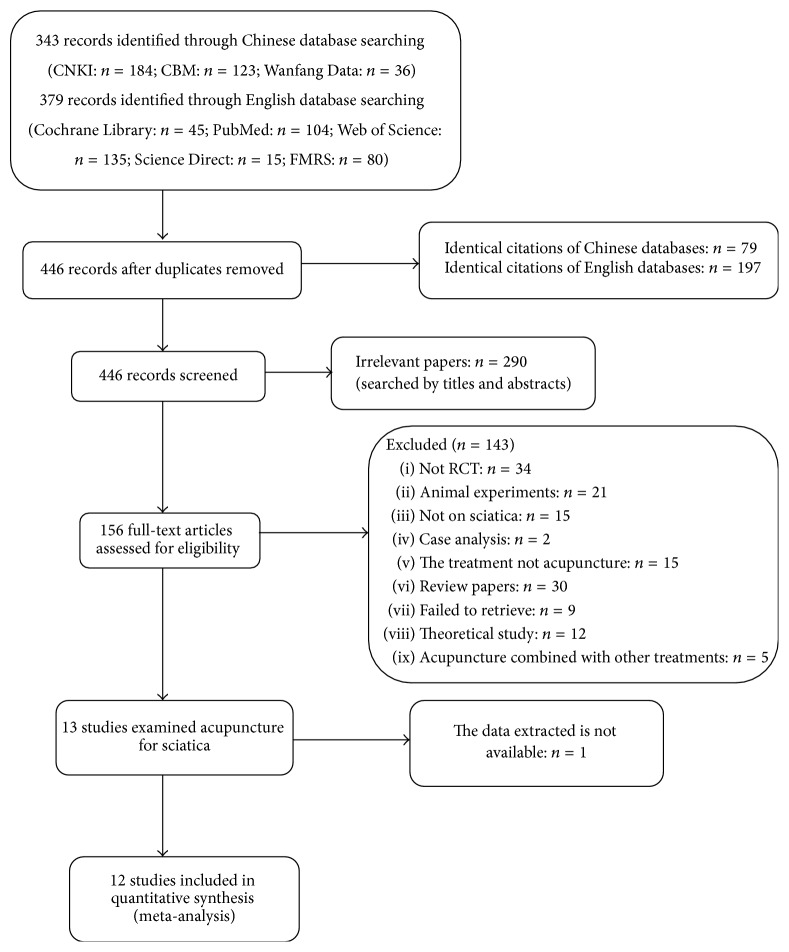
Flowchart of the trial selection process.

The database search obtained 722 records (343 records from Chinese databases and 379 records from English databases) potentially relevant to the research. Following removal of duplicates, 446 records remained (79 identical citations in Chinese and 197 identical citations in English). A total of 290 trials were excluded following reading of the titles and abstracts, due to lack of relevance. The full text of the remaining 156 articles was read and analyzed in detail, with 12 papers finally included for the systematic review. This screening process is summarized in a flow diagram (Figure 1).

### 3.2. Study Characteristics and Quality

All of the included trials originated in China, with a total of 1842 participants (901 in treatment groups and 941 in control groups). Of the 12 included studies, 11 demonstrated no significant difference at baseline in gender, age, and other basic information and the remaining study [10] did not report any information about the participants. Two studies [12, 19] have two control groups. Mean age ranged between 18.0 and 77.0 years and disease duration ranged from 4 days to 18 years. Uniformity of inclusion criteria was limited, with five studies [11, 15, 17, 20, 22] mentioning the type of sciatica (one with primary sciatica [15], two with secondary sciatica [17, 20], one with trunk-sciatica [11], and one with root sciatica [22]) and two studies [10, 22] not stating the duration of symptoms. The basic characteristics of the included trials are presented in Table 1.  Table 2 presents details of acupuncture treatment and control interventions of studies included in the meta-analysis.

**Figure 2:**
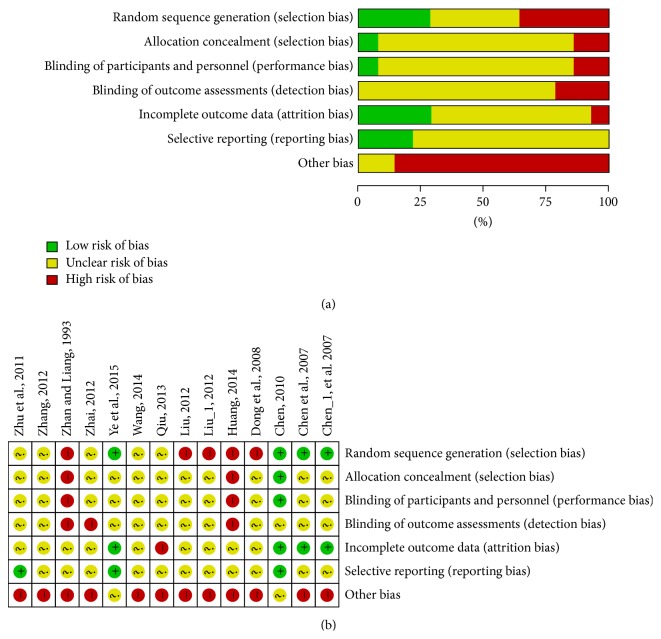
Quality assessment of included studies. (a) Risk of bias graph; (b) risk of bias summary.

For randomization, three studies [12, 16, 22] referred to a random digit table and one study [16] referred to a sealed envelope, with the remainder providing incomplete information. None of the studies were subject blinded or indicated whether the assessors were blinded to treatment allocation. The risk of bias assessment is shown in Figure 2.

### 3.3. Effectiveness

**Figure 3:**
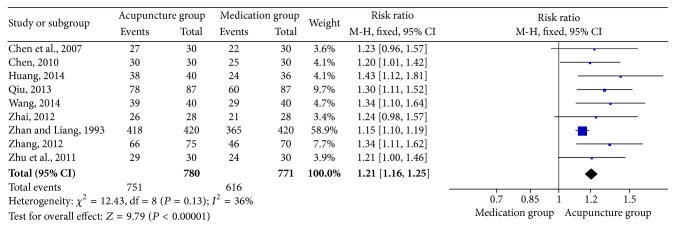
Forest of comparisons of total effectiveness between acupuncture group and medication group.

**Figure 4:**
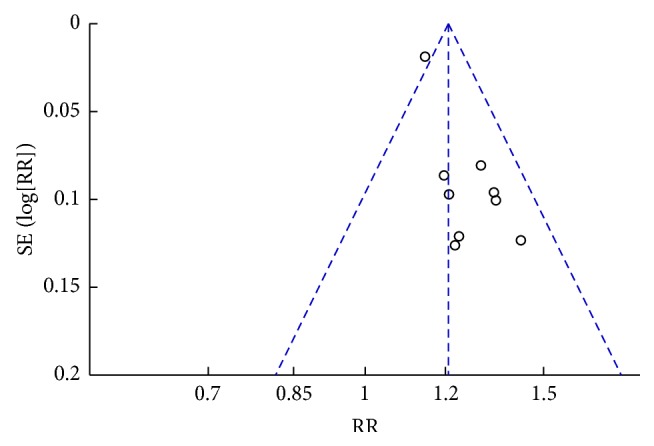
Funnel plot on effectiveness to evaluate the publication bias of the literatures.

Pooled analysis of nine studies [5, 10–12, 16–18, 20, 21] with 780 patients in the acupuncture group and 771 in the medication group revealed that acupuncture was significantly more effective than conventional medication (RR: 1.21; 95% CI: 1.16–1.25; *P* < 0.00001). As there was mild homogeneity in the consistency of the trial results (*χ* ^2^ = 12.43; *P* = 0.13; *I* ^2^ = 36%), a fixed effects model was applied (Figure 3). The graphic funnel plot of these nine studies appeared to be slightly asymmetric, suggesting the possibility of publication bias (Figure 4). In the subgroup analysis based on drug categories, subjects were divided into Nimesulide, Ibuprofen + Prednisone, Ibuprofen + Vitamin B1, and Indomethacin. The results did not change (Table 3). Sensitivity analysis was performed to assess the stability of the meta-analysis. When any single study was deleted, the corresponding pooled RR were changed slightly, with the statistically similar results indicating a good stability of the meta-analysis (Table 4).

### 3.4. Pain Intensity

**Figure 5:**
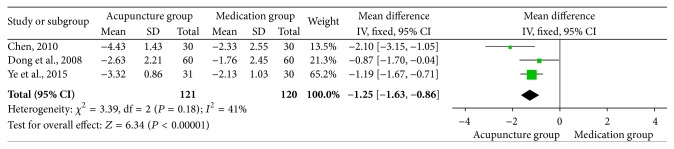
Forest of comparisons of pain intensity: acupuncture versus medication.

Three studies [15, 16, 22] reported pain intensity using a VAS to measure pain. The result revealed that acupuncture group experienced a significantly greater reduction in pain intensity than those who received conventional medication (MD: −1.25; 95% CI: −1.63 to −0.86; *P* < 0.00001). The result was homogenous (*χ* ^2^ = 3.39; *P* = 0.18; *I* ^2^ = 41%) and a fixed effects model was applied (Figure 5). In the subgroup analysis based on treatment method, subjects were divided into oral medication and external medication, and based on drug categories, subjects were divided into Ibuprofen + Prednisone, Ibuprofen, and Diclofenac. The results did not change (Table 3). The results also did not change in sensitivity analysis (Table 4).

### 3.5. Pain Threshold

**Figure 6:**
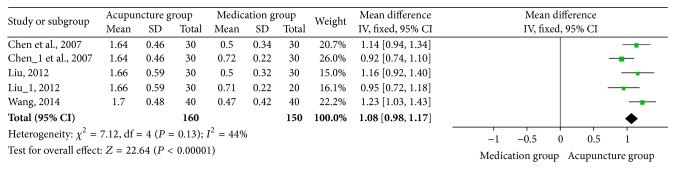
Forest of comparisons of pain threshold: acupuncture versus medication.

Pain threshold data were available in three studies [12, 19, 21] (two studies included two control groups). Meta-analysis revealed that acupuncture increased pain threshold favorably compared with medication (MD: 1.08; 95% CI: 0.98–1.17; *P* < 0.00001). The result was homogenous (*χ* ^2^ = 7.12; *P* = 0.13; *I* ^2^ = 44%) and a fixed effects model was applied (Figure 6). In the subgroup analysis based on treatment method, subjects were divided into oral medication and point-injection medication, and based on drug categories, subjects were divided into Nimesulide and 654-2. Change of the results was not found (Table 3). And similar results were also found in sensitivity analysis (Table 4).

### 3.6. Withdrawal and Adverse Effects

As shown in Table 1, only one trial [16] mentioned withdrawal, reporting no expulsion case. Three trials [16, 17, 19] mentioned adverse effects. Zhang [17] and Liu [19] reported no adverse effects of the acupuncture treatment. In the study of Chen [16], two cases with subcutaneous hemorrhage occurred after needling in the treatment group and the symptom of blood stasis disappeared after three or four days of hot pack.

## 4. Discussion

Sciatica is one of the most frequently reported complaints affecting quality of life and reducing social and economic efficiency. Most of this cost is not generated by medical treatment but attributable to loss of productivity [28]. In TCM, it is classified into the category of* Bi* syndrome (Bi Zheng) [29]. Conservative and surgical intervention are two predominant choices for therapy. Conservative treatment for sciatica is primarily aimed at pain reduction, either by analgesics or by reducing pressure on the nerve root, and includes prescription drugs, acupuncture, epidural steroid injections, spinal manipulation, traction therapy, hot packs, and muscle relaxants [30]. Surgical intervention focuses on eliminating the suspected cause of the sciatica, by the removal of part or all of the herniated disc and the alleviation of foraminal stenosis [30].

Acupuncture is an established adjuvant analgesic modality for the treatment of chronic pain, and it is considered a cure for many ailments and disorders [31]. The acupuncture-induced intricate feeling (soreness, numbness, heaviness, and distension) in the deep tissue beneath the acupoint is essential to acupuncture analgesia [32]. Acupuncture is thought to stimulate inhibitory nerve fibers for a short period, reducing transmission of pain signal to the brain [33]. Acupuncture treatment activates endogenous analgesic mechanisms [34], causing secretion of endorphin which is an endogenous opioid [35] and triggering release of adenosine [36], producing a rapidly effective analgesic action on radicular sciatica. Extensive research has shown that acupuncture analgesia may be initiated by stimulation of high-threshold, small-diameter nerves in the muscles [37]. A cohort study found that after electroacupuncture (EA) to the spinal nerve root, the symptoms of patients with radicular sciatica were immediately and markedly reduced [38]. Animal experiments have revealed that acupuncture is a better treatment for regeneration of crushed sciatic nerves than diclofenac sodium [39]. Data exists demonstrating that EA intervention can attenuate pain via regulation of expression of multiple proteins in the hypothalamus [40]. Thus, acupuncture is worthy of wide clinical application.

Following a comprehensive search of eight electronic databases, 12 studies (1842 participants) were included in the review. Analysis of outcomes revealed that acupuncture is more effective than medication for individuals with sciatica for effectiveness (RR 1.21; 95% CI: 1.16–1.25), pain intensity (MD −1.25; 95% CI: −1.63 to −0.86), and pain threshold (MD: 1.08; 95% CI: 0.98–1.17). The pooled results of this meta-analysis indicate that acupuncture is clinically effective, reduces pain intensity, and increases pain threshold in patients with sciatica compared with medication. In the subgroup analysis, the results did not change in different treatment method and drug categories. And in the sensitivity analysis, omitting the study of Zhan and Liang [10] in 1993 or Chen [16] in 2010, the heterogeneity changed from moderate to low. The reasons of the slight change might be that the design of the former one was not restricted (lacking basic data, such as patients characteristics, specific interventions) and the time of performing was too early and the medication routes of the latter were different from others.

Despite an extensive literature search, only a limited number of studies were available, hampering clear and exact conclusions. Most of the randomized controlled trials had low methodological quality with a high risk of bias. All selected trials demonstrated randomization; however, the processes of randomization and allocation concealment were not adequately described and blinding of patients and assessors was seldom mentioned. Only three trials [12, 16, 22] mentioned random sequence generation and only one demonstrated [16] allocation concealment, with none of the trials being blinded. Therefore, selection bias may have existed. For those studies without adequate explanation of quality control measures, it is difficult to rule out the possibility of selective bias, implementation bias, and measurement bias, which may lead to unreliable results. Except for methodological heterogeneity, there existed clinical diversity. (1) Variations of acupuncture: according to TCM, sciatica is caused by invasion of wind-cold or wind-damp, obstruction of channel due to blood stasis or stagnation [8]; based on TCM theory, all acupuncture procedures (e.g., points used, method of stimulation, and number of treatment sessions) need to be performed according to syndrome differentiation and individual differences, so acupuncture modalities are various from study to study and difficult to master and unify. For example, Qiu [5], Huang [20], Zhang [17], and Wang [21] selected acupuncture points based on different parts of pain and types of syndrome. A lack of TCM knowledge can reduce the therapeutic effect to some extent. Therefore, practisers are required to have a deep understanding of sciatica and acupuncture from the aspect of TCM so that clinical techniques could be rigorous [41]. (2) Criterion of CWM: although the CWM were categorized into NSAIDs, steroids, and vitamins, there still existed difference between Piroxicam, Ibuprofen, Nimesulide, and Indomethacin. Additionally, the duration and doses of administered drugs might influence the therapeutic effect to some extent. Above all, the appearance of clinical heterogeneity could be reasonably explained and further solved. Meanwhile, further methodologically robust trials are required. Therefore, although acupuncture may be effective in reducing pain and improving the symptoms compared to medication, the analysis results should be interpreted with caution.

It is evident that further studies of higher quality and with longer-term follow-up are needed for better quality and a more accurate analysis. To clarify the exact effect of acupuncture on patients with sciatica, further well-designed studies are needed. Further study design should take into account the following points: (1) the design should utilize strictly randomized, controlled, double-blind methods with patients selected objectively with standard eligibility; (2) all clinical studies of acupuncture should abide by STRICA; (3) appropriate sample size is required; (4) there is need for long-term follow-up; (5) there is consistency in the inclusion, exclusion, and diagnosis criteria; (6) there is implementation of standardized adverse event monitoring.

For sciatica, there is no gold standard for diagnosis and treatment so that it is difficult to establish effective form of treatment. Acupuncture is used to treat a variety of symptoms, especially pain, and has been demonstrated to be effective, safe, and well tolerated. From our meta-analysis, it is evident that acupuncture could be efficacious in treating the pain associated with sciatica. Although we were unable to draw definite conclusions due to the poor quality of the available trials, this positive result could provide clinicians with an accessible assessment of its therapeutic value and draw attention to acupuncture research.

## Figures and Tables

**Table 1:** Data summary and characteristics of the 12 studies included in meta-analysis.

Study ID	Group	Age (year)^*∗*^	Duration (month)^*∗*^	Sample size *N* (M/F)	Outcome	Type of sciatica	Diagnostic criteria	Withdrawal/adverse effects
Zhan and Liang, 1993 [10]	Acupuncture CWM	NA NA	NA NA	420 420	Effectiveness	NA	NA	NA/NA

Chen et al., 2007 [12]	Acupuncture CWM1 CWM2	34.24 ± 5.78 33.36 ± 7.58 35.78 ± 9.65	63 ± 43.08 69.36 ± 58.44 56.52 ± 47.52	30 (22/8) 30 (21/9) 30 (20/10)	Effectiveness pain threshold	NA	The clinical diagnostic and curative criteria of diseases (2nd) (1999)	NA/NA

Dong et al., 2008 [15]	Acupuncture CWM	25–64 22–65	0.57–252 0.3–216	60 (32/28) 60 (34/26)	Pain intensity	Primary sciatica	3200 standard diagnoses of diseases in internal medicine	NA/NA

Chen, 2010 [16]	Electroacupuncture CWM	41.72 ± 10.01 42.10 ± 9.87	25.29 ± 8.12 24.21 ± 7.98	30 (12/18) 30 (11/19)	Effectiveness pain intensity	NA	Criteria of diagnosis of diseases and syndromes in traditional Chinese medicine (1994) Practical diagnostics and therapeutics of integrated traditional Chinese and Western medicine (1995)	A/A

Zhu et al., 2011 [11]	Electroacupuncture CWM	18–75 19–76	1–60 1.5–66	40 (22/18) 40 (21/19)	Effectiveness	Trunk-sciatica	Physical examination, physical test (straight-leg-raising test), and diagnostic imaging	NA/NA

Zhang, 2012 [17]	Acupuncture CWM	35 ± 4.5	0.3–48	145 (89/56)	Effectiveness	Secondary sciatica	Physical examination, physical test (the sciatic nerve traction syndrome), and diagnostic imaging	NA/A

Zhai, 2012 [18]	Acupuncture CWM	22–71 22–70	0.33–12 0.1–12	28 (17/11) 28 (16/12)	Effectiveness	NA	Physical examination, physical test (straight-leg-raising test), and diagnostic imaging	NA/NA

Liu, 2012 [19]	Acupuncture CWM1 CWM2	29–35	60–108	80 (43/37)	Pain threshold	NA	The clinical diagnostic and curative criteria of diseases	NA/A

Qiu, 2013 [5]	Acupuncture CWM	24–68 25–69.5	0.4–60 0.5–72	87 (52/35) 87 (54/33)	Effectiveness	NA	Physical examination, physical test (the sciatic nerve traction syndrome), and diagnostic imaging	NA/NA

Huang, 2014 [20]	Acupuncture CWM	36 ± 4.6	10–48	76 (50/26)	Effectiveness	Secondary sciatica	Physical examination, physical test (the sciatic nerve traction syndrome), and diagnostic imaging	NA/NA

Wang, 2014 [21]	Electroacupuncture CWM	53.29 ± 3.17	63.84 ± 18.84	80 (43/37)	Effectiveness pain threshold	NA	The clinical diagnostic and curative criteria of diseases	NA/NA

Ye et al., 2015 [22]	Electroacupuncture CWM	58.2 ± 9.1 55.5 ± 7.1	NA NA	31 (12/19) 30 (11/19)	Pain intensity	Root sciatica	Criteria of diagnosis and therapeutic effect of diseases and syndromes in traditional Chinese medicine (1994) Guiding principle of clinical research on new drugs of traditional Chinese medicine (trial) (2002)	NA/NA

A: available; M: male; F: female; NA: not available; CWM: conventional Western medicine; RCT: randomized controlled trial.

^*∗*^Age and duration were shown in mean ± standard deviation or minimum–maximum.

**Table 2:** Details of acupuncture treatment and control interventions of studies included in the meta-analysis.

Study ID	Acupuncture rationale	Details of needling				Treatment regimen		Control interventions (drug/dosage/frequency)
		Points used	Depth of insertion/response sought	Needle stimulation	Needle type/retention time	No. TS	Frequency	
Zhan and Liang, 1993 [10]	TCM	Shenshu (BL 23), Zhishi (BL 53), Zhibian (BL 54), Huantiao (GB 30), Yanglingquan (GB 34), Xuanzong (GB 39), Qiuxu (GB 40), Taichong (LR 3), and Fengfu (GV 16)	NA/NA	Manual	Number 30 needles (Hua Tuo card)/20 min	40	NA	Prednisone, Vit B1, Piroxicam (taken orally)/NA/NA

Chen et al., 2007 [12]	TCM	Shenshu (BL 23), Dachangshu (BL 25), Huantiao (GB 30), Weizhong (BL 40), and Kunlun (BL 60)	NA/De qi	Manual	0.30 × 60–75 mm/5~30 min	10	Once a day, 10 times/course, and 3 days' rest after a course	C1: Nimesulide (taken orally)/0.10 g/bid C2: 654-2 injection (i.m.)/2 mg per acupoint/<10 mg/qd 10 times per course, 3 days' rest after a course

Dong et al., 2008 [15]	TCM	Huantiao (GB 30)	NA/Gaing of qi	Manual	0.3 mm × 4-inch unused sterile needles (Ruiqi Er brand)/30 min	15	Once a day, 15 times/course	Ibuprofen Sustained Release Capsules (taken orally)/300 mg/bid 15 times per course

Chen, 2010 [16]	TCM	Jiaji (L_2–4_) (EX-B2), Zhibian (BL 54), Huantiao (GB 30), Weizhong (BL 40), Chengshan (BL 57), Xuanzhong (GB 39), Kunlun (BL 60), Yinmen (BL 37), and Ashi point	2 inches for the points Zhibian and Yanglingquan, 3~4 inches for the point Huantiao; 1.5 inches for the points Yinmen, Weizhong, and Chengshan/muscle twitch	Electrical	0.30 × 25–40 mm/30 min	6	Three times per week, two weeks per course	Ibuprofen (taken orally)/0.2 g/tid; Prednisone (taken orally)/30 mg/tid 7 times per course

Zhu et al., 2011 [11]	TCM	Huantiao (GB 30), Juliao (GB 29), Weizhong (BL 40), and Chengshan (BL 57)	NA/Huantiao: Gaing of qi to toes	Electrical	NA/30 min	6	Once a day, 6 times/course, and 3 days' rest after a course	Ibuprofen (taken orally)/300 mg/bid; Vit B1 injection (i.m.)/100 mg/qd; Vit B12 injection (i.m.)/0.25 mg/qd 6 times per course, 3 days' rest after a course

Zhang, 2012 [17]	TCM	Posterior limb of lower limbs or back pain: Dachangshu (BL 25), Chengshan (BL 57), Huantiao (GB 30), and Weizhong (BL 40); lateral of lower limbs or buttocks pain: Huantiao (GB 30), Xuanzhong (GB 39), Yanglingquan (GB 34), and Fenglong (ST 40); all pain referred to above: Dachangshu (BL 25), Huantiao (GB 30), Zhibian (BL 54), Kunlun (BL 60), and Yanglingquan (GB 34)	NA/De qi	Manual	NA/30 min	6	Once a day, six times per week	Ibuprofen (taken orally)/0.6 g/tid; Prednisone/10 mg/tid 7 times per course, 2 days' rest after a course

Zhai, 2012 [18]	TCM	Dachangshu (BL 25), Shenshu (BL 23), Huantiao (GB 30), Weizhong (BL 40), and Kunlun (BL 60)	NA/De qi	Manual	0.30 × 60–75 mm/15~30 min	10	Once a day, 10 times/course, and 3 days' rest after a course	Nimesulide (taken orally)/0.10 g/bid 10 times per course, 3 days' rest after a course

Liu, 2012 [19]	TCM	Shenshu (BL 23), Dachangshu (BL 25), Huantiao (GB 30), Weizhong (BL 40), and Kunlun (BL 60)	NA/De qi	Manual	0.30 × 60–75 mm/NA	10	Once a day, 10 times/course, and 3 days' rest after a course	C1: Nimesulide (taken orally)/0.10 g/bid C2: 654-2 injection (i.m.)/2 mg per acupoint/<10 mg/qd 10 times per course, 3 days' rest after a course

Qiu, 2013 [5]	TCM	Lateral leg or buttocks pain: Xuanzhong (GB 39), Yanglingquan (GB 34), Huantiao (GB 30), and Fenglong (ST 40); back or posterior limb of lower limbs pain: Dachangshu (BL 25), Chengshan (BL 57), Huantiao (GB 30), and Weizhong (BL 40); all pain referred to above: Zhibian (BL 54), Kunlun (BL 60), Yanglingquan (GB 34), Dachangshu (BL 25), and Huantiao (GB 30)	NA/De qi	Manual	Number 26 6-inch long needles/30 min	10~12	Once a day, 5~6 times per week, and two weeks per course	Indomethacin (taken orally)/30 mg/bid; Vit B12 (i.m.)/500 u/qd

Huang, 2014 [20]	TCM	Lateral of lower limbs or buttocks pain: Yanglingquan (GB 34), Xuanzhong (GB 39), and Fenglong (ST 40); posterior limb of lower limbs or back pain: Chengshan (BL 57), Dachangshu (BL 25), and Huantiao (GB 30); all pain referred to above: Yanglingquan (GB 34), Zhibian (BL 54), Kunlun (BL 60), and Huantiao (GB 30)	NA/NA	Manual	NA/30 min	6	Once a day, six times per week	Ibuprofen (taken orally)/0.6 g/tid; Prednisone (taken orally)/10 mg/tid 7 times per course, 2 days' rest after a course

Wang, 2014 [21]	TCM	Main points: Zhibian (BL 54), Huantiao (GB 30), Yanglingquan (GB 34), Juliao (GB 29), Kunlun (BL 60), Chengshan (BL 57), and Xuanzhong (GB 39) Optional points: damp-heat syndrome: Yinlingquan (SP 9), Pishu (BL 20); cold-dampness syndrome: Yaoyangguan (GV 3), Shenshu (BL 23); blood stasis syndrome: Weizhong (BL 40), Ciliao (BL 32); deficiency of both qi and blood: Geshu (BL 17), Sanyinjiao (SP 6), and Zusanli (ST 36)	NA/De qi	Electrical	NA/30 min	10	Once a day, 10 times/course, and 5 days' rest after a course	Nimesulide (taken orally)/0.10 g/bid 10 times per course, 3 days' rest after a course

Ye et al., 2015 [22]	TCM	Jiaji (L4-5) (EX-B2), Jiaji (L5-S5) (EX-B2), Zhibian (BL 54), and Huantiao (GB 30)	NA/De qi	Electrical	NA/30 min	6	Two times per week, for three weeks	Diclofenac Diethylamine gel (external use)/4 g four times per day, three weeks

C: Control group; NA: not available; TCM: traditional Chinese medicine; No. TS: number of treatment sessions.

i.m.: intramuscular route; qd: once a day; bid: twice a day; tid: three times a day.

**Table 3:** The results of subgroup meta-analysis.

Subgroup	Eligible studies	Acupuncture group (number)	Medication group (number)	RR/MD (95% CI)	*P* value	Heterogeneity test	Effect model
*Effectiveness *							
Drug categories							
Nimesulide	3	98	98	1.28 (1.12, 1.45)	0.0002	*P* = 0.81, *I* ^2^ = 0%	Fixed
Ibuprofen + Prednisone	3	145	136	1.32 (1.17, 1.49)	<0.00001	*P* = 0.42, *I* ^2^ = 0%	Fixed
Ibuprofen + Vitamin B1	2	450	450	1.15 (1.11, 1.19)	<0.00001	*P* = 0.59, *I* ^2^ = 0%	Fixed
Indomethacin	1	87	87	1.30 (1.11, 1.52)	0.001	—	—

*Pain intensity*							
Treatment method							
Oral	2	90	90	−1.44 (−2.65, −0.24)	0.02	*P* = 0.07, *I* ^2^ = 69%	Random
External	1	31	30	−1.19 (−1.67, −0.71)	<0.00001	*—*	—
Drug categories							
Ibuprofen + Prednisone	1	30	30	−2.10 (−3.15, −1.05)	<0.00001	*—*	—
Ibuprofen	1	60	60	−0.87 (−1.70, −0.04)	0.04	*—*	—
Diclofenac	1	31	30	−1.19 (−1.67, −0.71)	<0.00001	*—*	—

*Pain threshold*							
Treatment method							
Oral	3	100	100	1.18 (1.06, 1.30)	<0.00001	*P* = 0.81, *I* ^2^ = 0%	Fixed
Injection	2	60	50	0.93 (0.79, 1.07)	<0.00001	*P* = 0.81, *I* ^2^ = 0%	Fixed
Drug categories							
Nimesulide	3	100	100	1.18 (1.06, 1.30)	<0.00001	*P* = 0.81, *I* ^2^ = 0%	Fixed
654-2	2	60	50	0.93 (0.79, 1.07)	<0.00001	*P* = 0.81, *I* ^2^ = 0%	Fixed

RR: risk ratio; MD: mean difference; 95% CI: 95% confidence interval; 654-2: anisodamine.

**Table 4:** The results of the included studies through sensitivity analysis.

Excluded study	Acupuncture group (number)	Medication group (number)	RR/MD (95% CI)	*P* value	Heterogeneity test	Effect model
*Effectiveness*						
Before excluding	780	771	1.21 (1.16, 1.25)	*P* < 0.00001	*P* = 0.13, *I* ^2^ = 36%	Fixed
Chen, 2007	750	741	1.21 (1.16, 1.25)	*P* < 0.00001	*P* = 0.09, *I* ^2^ = 43%	Fixed
Chen, 2010	750	741	1.21 (1.16, 1.25)	*P* < 0.00001	*P* = 0.09, *I* ^2^ = 44%	Fixed
Huang, 2014	740	735	1.20 (1.15, 1.24)	*P* < 0.00001	*P* = 0.24, *I* ^2^ = 24%	Fixed
Qiu, 2013	693	684	1.20 (1.15, 1.24)	*P* < 0.00001	*P* = 0.19, *I* ^2^ = 30%	Fixed
Wang, 2014	740	731	1.20 (1.15, 1.25)	*P* < 0.00001	*P* = 0.18, *I* ^2^ = 31%	Fixed
Zhai, 2012	752	743	1.21 (1.16, 1.25)	*P* < 0.00001	*P* = 0.09, *I* ^2^ = 43%	Fixed
Zhang, 1993	360	351	1.29 (1.20, 1.39)	*P* < 0.00001	*P* = 0.93, *I* ^2^ = 0%	Fixed
Zhang, 2012	705	701	1.20 (1.15, 1.24)	*P* < 0.00001	*P* = 0.21, *I* ^2^ = 27%	Fixed
Zhu, 2011	750	741	1.21 (1.16, 1.25)	*P* < 0.00001	*P* = 0.09, *I* ^2^ = 44%	Fixed
*Pain intensity*						
Before excluding	121	120	−1.25 (−1.63, −0.86)	*P* < 0.00001	*P* = 0.18, *I* ^2^ = 41%	Fixed
Chen, 2010	91	90	−1.11 (−1.53, −0.70)	*P* < 0.00001	*P* = 0.51, *I* ^2^ = 0%	Fixed
Dong, 2008	61	60	−1.52 (−2.38, −0.66)	0.00005	*P* = 0.12, *I* ^2^ = 58%	Random
Ye, 2015	90	90	−1.44 (−2.65, −0.24)	0.02	*P* = 0.07, *I* ^2^ = 69%	Random
*Pain threshold*						
Before excluding	160	150	1.08 (0.98, 1.17)	*P* < 0.00001	*P* = 0.13, *I* ^2^ = 44%	Fixed
Chen, 2007	130	120	1.06 (0.91, 1.22)	*P* < 0.00001	*P* = 0.08, *I* ^2^ = 55%	Random
Chen_1, 2007	130	120	1.13 (1.02, 1.24)	*P* < 0.00001	*P* = 0.34, *I* ^2^ = 11%	Fixed
Liu, 2012	130	120	1.06 (0.91, 1.21)	*P* < 0.00001	*P* = 0.09, *I* ^2^ = 54%	Random
Liu_1, 2012	130	130	1.11 (0.96, 1.25)	*P* < 0.00001	*P* = 0.12, *I* ^2^ = 48%	Fixed
Wang, 2014	120	110	1.03 (0.91, 1.16)	*P* < 0.00001	*P* = 0.25, *I* ^2^ = 27%	Fixed

RR: risk ratio; MD: mean difference; 95% CI: 95% confidence interval.
